# Design and Characterization of a Transcriptional Repression Toolkit for Plants

**DOI:** 10.1021/acssynbio.4c00404

**Published:** 2024-09-24

**Authors:** Kasey Markel, Jean Sabety, Shehan Wijesinghe, Patrick M. Shih

**Affiliations:** † Department of Plant and Microbial Biology, University of California, Berkeley, California 94720, United States; ‡ Feedstocks Division, Joint BioEnergy Institute, Emeryville, California 94608, United States; § Environmental Genomics and Systems Biology Division, Lawrence Berkeley National Laboratory, Berkeley, California 94608, United States; ∥ Department of Plant Biology, University of California, Davis, California 95616, United States; # Innovative Genomics Institute, University of California, Berkeley, California 94720, United States

**Keywords:** plant biology, plant synthetic biology, transcriptional repression, protein function prediction, tool development

## Abstract

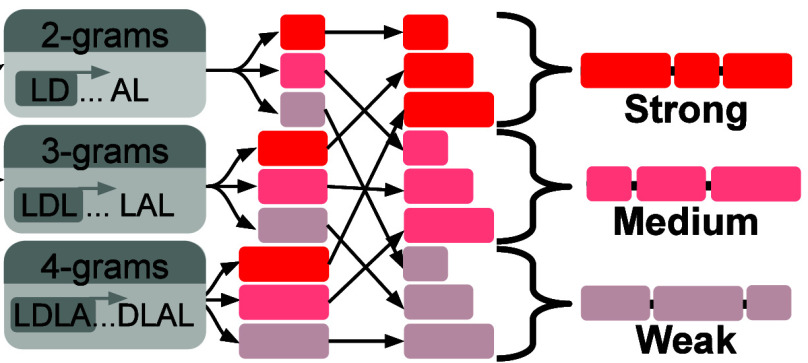

Regulation of gene expression is essential for all life. Tools to manipulate the gene expression level have therefore proven to be very valuable in efforts to engineer biological systems. However, there are few well-characterized genetic parts that reduce gene expression in plants, commonly known as transcriptional repressors. We characterized the repression activity of a library consisting of repression motifs from approximately 25% of the members of the largest known family of repressors. Combining sequence information with our trans-regulatory function data, we next generated a library of synthetic transcriptional repression motifs with function predicted in advance. After characterizing our synthetic library, we demonstrated not only that many of our synthetic constructs were functional as repressors but also that our advance predictions of repression strength were better than random guesses. Finally, we assessed the functionality of known transcriptional repression motifs from a wide range of eukaryotes. Our study represents the largest plant repressor motif library experimentally characterized to date, providing unique opportunities for tuning transcription in plants.

## Introduction

Tuning of expression levels of genes is among the most powerful methods for controlling the phenotype of a cell and is extensively used by both nature and biotechnologists.^1−3^ For plants in particular, the modulation of the expression of existing genes has led to some of the greatest successes in the history of plant biotechnology, such as reduced browning in apples^2^ and resistance to viruses *via* silencing of viral genes.^4^ Despite these standout successes, plant biotechnologists have relatively few tools for gene expression modulation, which limits our capacity to engineer plants.

One highly customizable approach for controlling plant phenotype involves targeted repression of the genes encoding pathway enzymes that result in metabolites not desired in a particular tissue type or developmental time point. Because transcriptional repression is mediated by proteins, this approach allows for spatial and temporal control of pathway flux through the application of tissue-, developmental-stage-, or physiological-state-specific promoters to drive transgenes encoding the repressor proteins. A substantial amount of research has been invested in the development of RNA-guided dead nuclease-mediated activation, but less research has focused on repression. Of the existing repression systems, a key limitation is a lack of diversity of well-characterized repression motifs.

The largest known family of repression motifs in plants are the ethylene-responsive element binding-factor associated amphiphilic repression (EAR) domains, characterized by the canonical motifs LxLxL or DLNxxP, where x can be any amino acid. EAR-motif-containing proteins have substantially increased in number in the lineage leading to land plants and constitute 0.5–2% of protein-encoding genes in angiosperms.^5^ The mechanism of EAR-mediated repression is not entirely understood but is at least in part due to binding with the TPL corepressor, which in turn recruits histone deacetylases.^6^ The best-characterized EAR repression motif is SRDX, a 12 amino acid motif identified through mutational modifications of the repression domain from the *Superman* transcription factor.^7^ While this tool has proven useful for modulation of hormone signaling,^8^ improving salt tolerance,^9^ and inducing male sterility for breeding,^10^ it is not suitable for all transcriptional repression projects because it only offers one level of downtuning. Here we aimed to expand the range of characterized repression motifs to enable plant synthetic biology applications.

## Results and Discussion

### Characterization of Natural EAR Repression Motifs

**Figure 1:**
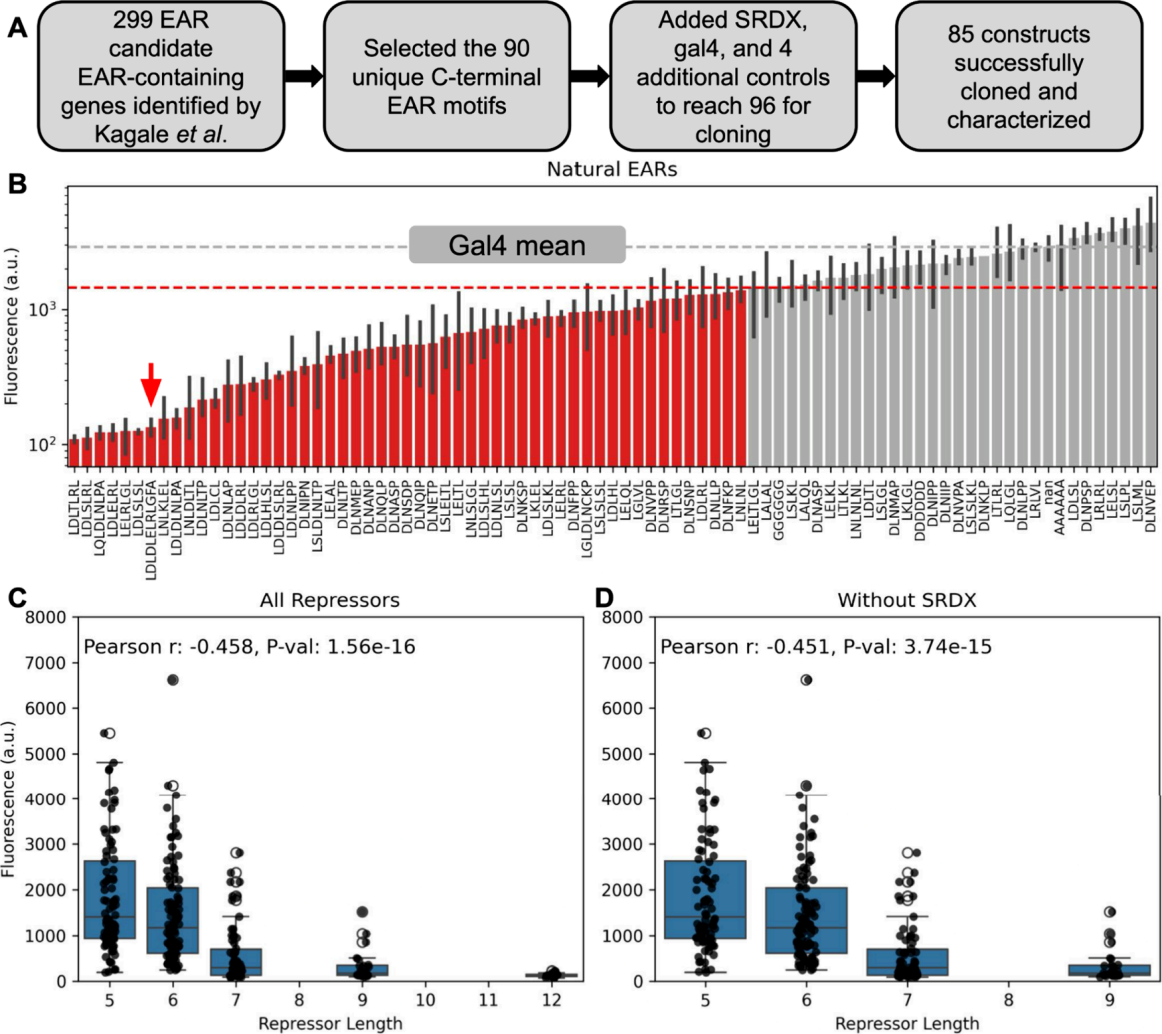
**Natural EAR repression motifs span a broad range of activity, and repression motif length correlates with strength.** (A) Flowchart of our selection and cloning of candidate EAR repression motifs. (B) Fluorescence data from the repressor assay. Constructs in gray display minimal activity, and constructs in red are repressors. Horizontal lines indicate mean activity of Gal4 (gray line) and the threshold for a construct being classified as a repressor (red line). The red arrow indicates SRDX, and error bars indicate the standard error of the mean (SEM). (C) Strength of repression motifs by length, measuring all constructs in this library. (D) Strength of repression motifs by length with SRDX, which is one of the strongest constructs and the longest one, removed. Boxes indicate 25th, 50th, and 75th percentile. Raw data are plotted as points.

We initially mined a previously published bioinformatic search of the *Arabidopsis* genome for putative EAR repressors.^11^ From this list, we identified all putative EAR repression motifs located on the C-terminal end of the protein, which we believed might be most accurately assessed in a medium-throughput assay that depends on C-terminal fusions of trans-elements.^12^ In brief, our assay relies upon fusing each trans-element on the C-terminus of the well-characterized Gal4 DNA-binding domain, which acts in concert with a green fluorescent protein (GFP) driven by a promoter which contains a Gal4 binding site, as has been previously described.^12,13^ The fluorescence of GFP thereby enables us to determine the trans-regulatory behavior of the putative repression motif. We identified 90 unique EAR motifs in that set and added SRDX and Gal4 alone as key controls: a positive control for a strong known repression motif and a negative control to indicate the baseline GFP fluorescence level in the absence of a repression motif. We successfully cloned fusions of 84 EAR motifs to Gal4 and infiltrated them one by one into *Nicotiana benthamiana* leaves for analysis (see Figure 1A).

The majority of the putative EAR motifs fused to DNA-binding regions indeed function as repressors in this system, validating the bioinformatic analysis of Kagale *et al.*(11) (Figures 1B and S1; data in Table S1). We found that 53 of our 84 constructs passed our threshold of 50% GFP reduction to be classified as repressors. The constructs covered a wide dynamic range from a 51% increase to a 96% reduction of GFP expression. These repressors cover a wide range of repression activity from barely distinguishable from the negative control up to somewhat stronger than SRDX, the strongest previously described plant transcriptional repression motif. While some repressors had stronger activity than SRDX in our screen, a Dunn test with Bonferroni correction for multiple comparison revealed the differences to not be statistically significant (see the Supplemental Code on GitHub). When we compared the lengths of the repression motifs to their average repression strengths, we discovered that longer repression motifs were on average stronger than shorter ones (Figure 1C). This effect is not an artifact of SRDX’s effect as the longest and one of the strongest repression motifs; the correlation dropped only a minor amount when we removed it from the dataset (Figure 1D). We also discovered that constructs containing both the DLNxxP and LxLxL canonical motifs were more likely to act as repressors: of the six constructs containing both canonical motifs, all were repressors and all were in the stronger half of the constructs, a statistically significant enrichment (Fisher’s exact test *P* = 0.0276; Figure S1).

### A Repression Motif Can Convert an Activating Transcription Factor into a Repressor in *Arabidopsis* Stable Lines

**Figure 2:**
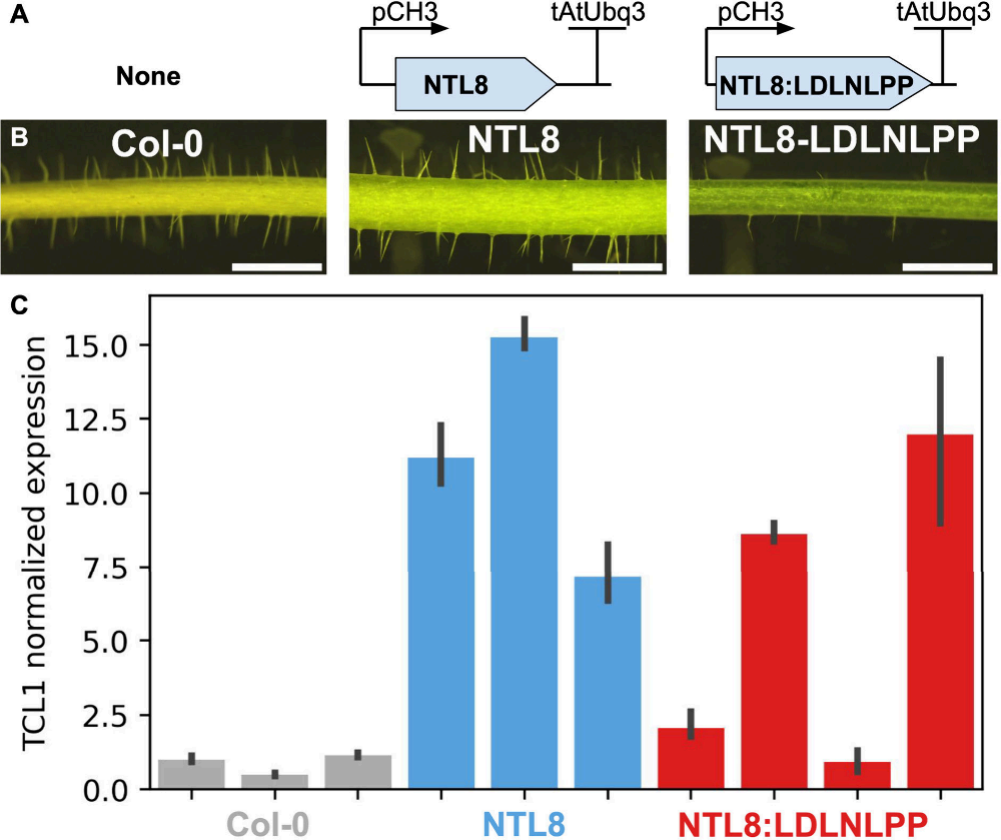
**NTL8-repression motif fusions can modulate trichome density**. (A) Details of the constructs used for *Arabidopsis* stable-line transformation. (B) Images of the floral stem trichome density on T0 stably transformed plants. Scale bars = 2 mm. (C) RTqPCR data for the expression level of TCL1 normalized by Ef1α, with the average Col-0 expression set to 1. Primer sequences are available in Table S2, and data are available in Table S3. Error bars indicate the SEM.

To verify the activity of these repression motifs in a more natural context, we fused one of our EAR repression motifs to an activating transcription factor to convert it into a repressor, as has been previously demonstrated with SRDX.^8^ We performed this experiment in stably transformed *Arabidopsis thaliana*. For a proof-of-concept test-case transcription factor we selected NTL8, a regulator of trichome development.^14^ We made a fusion of NTL8 and the natural EAR repression motif LDLNLPP driven under the constitutive promoter pCH3 (At4G13930)^15^ (Figure 2A). As expected, overexpression of NTL8 seemed to result in qualitatively higher trichome density, whereas NTL8–LDLNLPP resulted in qualitatively lower trichome density (Figure 2B). For a quantitative readout, we performed RTqPCR on TCL1, which is activated by wild-type NTL8.^14^ While there was substantial variation in expression levels between independent lines, NTL8–LDLNLPP appears to have a lower expression of TCL1 than NTL8 alone (Figure 2C; primer sequences in Table S2, data in Table S3). Much of the variance in TCL1 expression between independent lines is explained by different levels of transgenic NTL8 expression, presumably as a result of insertional effects (Figure S2). This confirms not only that repression motifs function in a different species from the one in which they were initially characterized but also that they are capable of modulating the activity of transcription factors and therefore tuning natural gene regulatory networks.

### Design and Characterization of Synthetic EAR Repression Motifs

**Figure 3:**
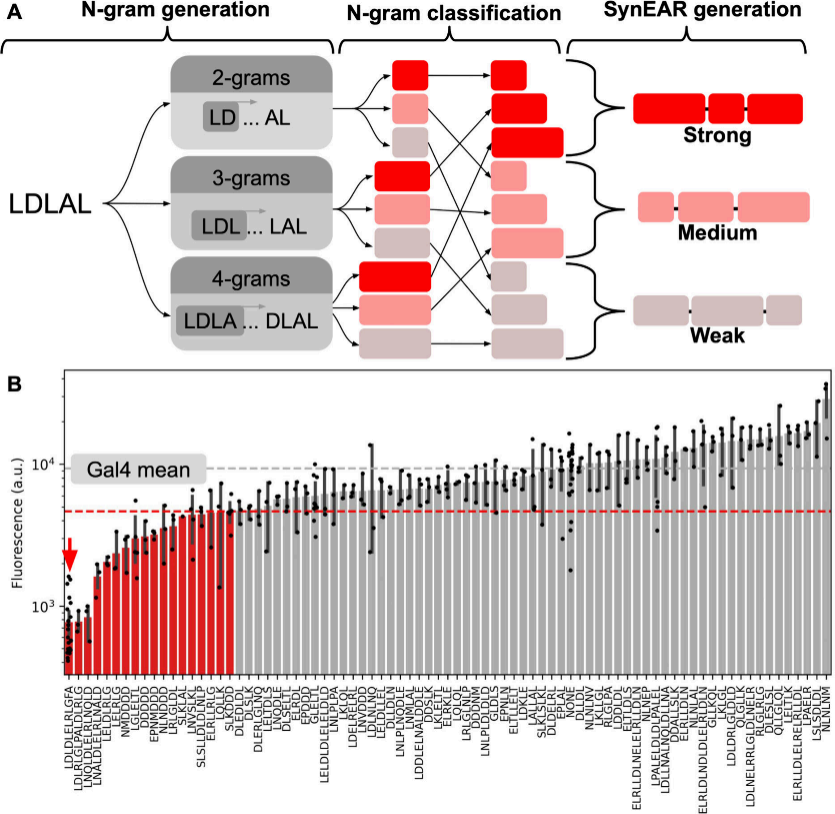
**Natural language processing model facilitates generation of SynEAR repression motifs.** (A) Cartoon diagram of the *n*-gram-based process used to generate SynEARs. The process is depicted from left to right. (B) Repression performance data of SynEARs. The gray dashed line indicates the mean value of Gal4, and the red line indicates the threshold for repressors. The red arrow indicates SRDX. Error bars indicate the SEM, and points indicate raw data.

In an effort to further understand the relationship between the sequence composition of the EAR repression motifs and their transcriptional trans-regulatory activity, we applied the *n*-gram analysis method first developed for natural language processing,^16^ which has more recently been successfully applied to analyze DNA^17^ and protein^18^ sequences. This method breaks down linear sequences—like words or amino acids—into small “grams” of various integer lengths *n* and thereby can encode not just the relative frequencies of amino acids but also the spatial relationships. As depicted in Figure 3A, all sequences were decomposed into all possible 2, 3, and 4 amino acid long *n*-grams. These *n*-grams were then sorted into three categories: those overrepresented among the strongest repressor constructs, those without any over-representation, and those over-represented among the weakest constructs (see Materials and Methods for detailed thresholds; all *n*-grams in each category are available in Table S4). These *n*-grams were then randomly concatenated within the length distribution of the original EAR library to create a set of synthetic EAR (SynEAR) constructs. SynEARs were generated in four predicted categories of strength: weak, moderate, strong, and strongest. The first three were constructed of their respective associated *n*-grams with the same length distribution as the natural EAR library, while the “strongest” was generated from strong *n*-grams with a longer length distribution in light of the finding that natural EAR repression strength is correlated with length (sequences of all SynEARs are available in Table S5).

These SynEARs were cloned and characterized *in planta* in a fashion similar to that for the natural EAR library (Figure 3B; data in Table S5). Despite being composed of *n*-grams from EAR repression motifs, a smaller fraction of the SynEARs (only 18 of 80) met our threshold for clear repression (Figure 3B). We suspect that this overall lower repression activity of the SynEAR library compared to the natural EAR library is due to a failure to recapitulate the EAR canonical motifs, which are larger than the length of our longest *n*-grams. Indeed, the majority of SynEARs contain neither of the two classic EAR canonical motifs (Figure S3), highlighting a shortcoming in this method for the generation of synthetic repressor constructs. Nonetheless, this SynEAR library did generate and characterize many synthetic trans-regulatory elements, some of which are statistically indistinguishable in repression strength from SRDX, the strongest previously characterized repression motif, despite having significantly different sequences, with all pairwise identities for statistically indistinguishable repression motifs under 45% (see the Supplemental Code for calculations).

### Evaluation of Predicted Function of SynEARs

**Figure 4:**
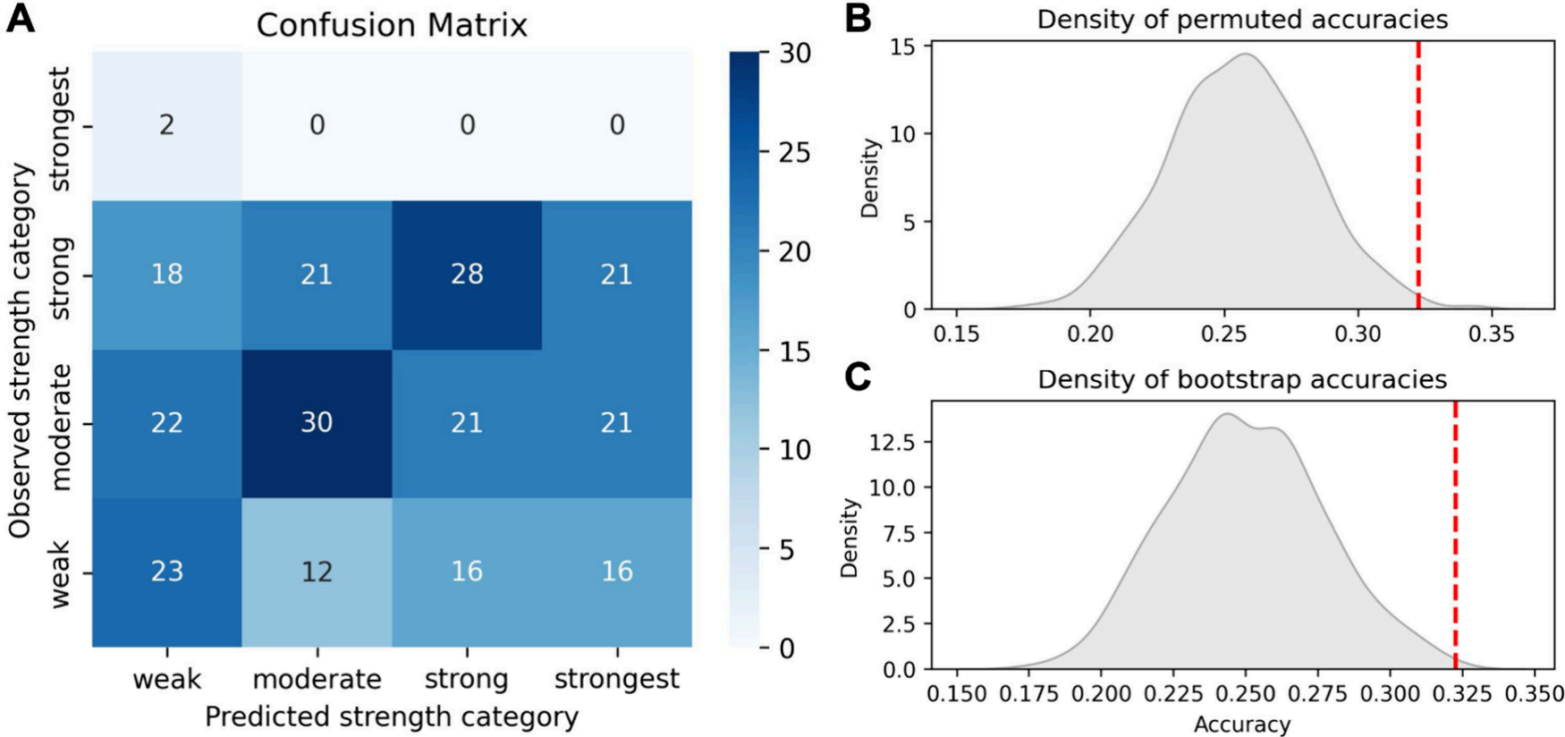
**Predictions of the activity of synthetic repression motifs are better than chance.** (A) Confusion matrix of predicted vs actual activity strength class. Numbers in the heatmap are the numbers of constructs with that combination of observed and predicted strengths. (B) Model accuracy robustness check comparing our model’s accuracy (red line) to 1000 randomly permuted model predictions. (C) Model accuracy robustness check comparing our model’s accuracy (red line) to 1000 randomly generated models with randomized bootstrap predictions.

Given that we generated and characterized this library of SynEARs, we next sought to determine whether the measured trans-regulatory activity matched our advance predictions. From simply plotting the SynEARs by predicted strength class (Figure S3A), it was clear that the prediction was not overwhelmingly accurate. Nonetheless, we were interested in whether the predictions were better than random guesses and settled on using the confusion matrix method, which among other use cases is widely used in assessment of classification systems.^19^ A confusion matrix is a table or heatmap in which predicted categories are plotted against observed real categories; a perfect classifier only has nonzero values at the *y* = *x* diagonal. In this case, the four predicted categories of SynEAR trans-regulatory activity were plotted against the actual repression level. Since the repression level is a continuous numerical value, it was necessary to establish thresholds to bin our numerical data into four categories of observed repression activity. We determined optimized thresholds in an unbiased fashion through an iterative approximation algorithm implemented in the Python package scikit-learn^20^ (see the Supplemental Code for precise implementation details). The resulting confusion matrix is shown in Figure 4A.

Our model prediction accuracy was 32.3%. To determine whether this was better than chance, we randomly shuffled the labels of the data and ran 1000 permutations of modeling using Python (see the Supplemental Code). These 1000 randomly shuffled versions of our predicted strength levels had an average accuracy of 25.7% with a standard deviation of 2.49%. Our actual model outperformed 99.4% of these shuffled permuted models (Figure 4B), for a two-tailed *p* value of 0.013 for the hypothesis that our model is either significantly better or significantly worse than a randomly shuffled version of our model’s guesses. As an additional robustness check, we compared our model against 1000 bootstrapped runs of a random guessing model. Our model accuracy of 32.3% compared well to the 25.0% mean performance from 1000 bootstrapped runs of random choice, with a standard deviation of 2.66% (Figure 4C). Our model performed 2.66 standard deviations above the mean for random guessing, meaning we outperformed 99.6% of random-choice models for a two-tailed *p* value of 0.0073 for the hypothesis that our model is statistically different from a randomly selected model.

### Characterization of Diverse Repression Motifs from Distant Eukaryotic Lineages

Having characterized a library of natural EARs and SynEARs, we next turned to other classes of repression motifs, including representative members from other families of plant repression motifs such as the B3 family^21^ as well as known nonplant eukaryotic repression motifs such as the *Drosophila* Hairy,^22^ Knirps,^23^ and Runt^24^ repression motifs (full list, data, and amino acid sequences are available in Table S6). We characterized these repression domains in a fashion similar to that for the EAR and SynEAR libraries and rather surprisingly found that the majority of these motifs when fused to DNA binding domains act as repressors in plants, despite substantial phylogenetic distance (Figure S4A). However, all tested non-EAR repression motifs had substantially lower repression activity than SRDX.

### Analysis of Tandem Concatenation of Repression Motifs

Since concatenation of genetic parts often results in an increase in their functionality,^25^ we also tested concatenated fusions of some of our previously characterized EAR and SynEAR repression motifs. All but one of these retained repression activity (Figure S4B; data in Table S6). However, none of these fused repression motif constructs was stronger than the strongest nonfused candidates, some of which were contained as components. Surprised by this finding that concatenation resulted in weaker overall repression level than the component parts, we devised a simpler experiment comparing single, double, and triple tandem repeats of SRDX, following a previous report which tested 3×SRDX but did not compare it to single or doubly repeated versions.^26^ To our surprise, SRDX and 2×SRDX displayed statistically indistinguishable levels of repression, while 3×SRDX was noticeably weaker (Figure S5; data in Table S7). This suggests that concatenation of repression motifs does not reliably increase repression strength and may even weaken it.

## Conclusion

With limited exceptions, the genome sequences of every cell of a given organism are identical. The massive variation in cellular structure, metabolism, and function between different tissue types within an organism is primarily generated through differences in the expression levels of genes. Our set of transcriptional repression motifs enables more fine-grained control of the expression level of plant genes when expression levels lower than the natural baseline are required. These repressors will also likely function as targetable repressors when fused to catalytically dead RNA-guided endonucleases, as SRDX has already been shown to do,^26^ enabling a wide dynamic range of gene expression tuning.

Ideally, it would be possible to compare the activities of all parts within a transcription expression tuning toolkit. To facilitate comparisons between repressor constructs tested in different datasets, we normalized all constructs within each of the three libraries by their two shared common elements, Gal4 and SRDX (Table S8). While there are inherent limitations in making comparisons between constructs tested as parts of different libraries, this dataset will serve as a simple combined resource to compare the approximately 200 constructs characterized in this study.

To our knowledge, this is the most comprehensive characterization of plant transcriptional repression motifs to date, with a particular focus on short repressor motifs, which are especially useful for plant engineering and synthetic biology efforts where length can be a restriction. Indeed, these motifs are short enough that they can be cloned as primer tails, simplifying plasmid construction and enabling higher-throughput testing. These repression motifs have broad value for plant synthetic biology, including applications such as the repression of native genes, modulation of the function of native transcription factors, or construction of synthetic genetic circuits. We hope that this transcriptional repression toolkit will enable more precise control of the gene expression level in engineered plants.

## Materials and Methods

### Plant Growth Conditions

*Nicotiana benthamiana* was grown following a previously published lab protocol.^15^ Plants were grown in an indoor growth room at 25 °C and 60% humidity using a 16 h/8 h light/dark cycle with a daytime PPFD of ∼120 μmol m^–2^ s^–1^. Soil consisted of Sunshine Mix #4 (Sungro) supplemented with Osmocote 14–14–14 fertilizer (ICL) at 5 mL/L and agroinfiltrated 29 days after seed sowing. *Arabidopsis thaliana* Col-0 were germinated and grown in Sunshine Mix #1 soil (Sungro) in a Percival growth chamber at 22 °C and 60% humidity using an 8 h/16 h light/dark cycle with a daytime PPFD of ∼200 μmol m^–2^ s^–1^.

### *N. benthamiana* Agroinfiltration Assay

Each construct was infiltrated in conjunction with the GFP reporter into one leaf per plant and three plants per construct. For each leaf, eight technical replicates of leaf disk were removed for analysis on a plate reader, and the data were averaged to form one biological data point.

Tobacco agroinfiltration was performed according to a previously published standardized lab method,^12^ with the minor modification that a reporter construct with a stronger GFP promoter was used (pSynUAS19_WUS instead of pSynUAS17_WUS) in order to optimize the dynamic range of the assay for repressors. Fluorescence values recorded on an Omega Biotek plate reader were obtained for eight leaf disks per plant, and those technical replicate values were averaged to generate the by-biological-replicate values available in the supporting tables.

### Transformation of *A. thaliana*

Floral dip transformation was performed according to a previously described protocol.^27^ Seeds from floral dipped plants were selected on 50 mg/L kanamycin in plates sealed with micropore tape and a plate composition of 1.5% plant TC agar, 1/2 MS with nutrients at pH 5.6. Seedlings were allowed to grow for 2 weeks in a Percival growth chamber dedicated to axenic plant growth with constant light at 24 °C. After 2 weeks, transformant and nontransformant plants were easily differentiable *via* size (transformants larger) and cotyledon color (bleached yellow versus green). At that point, transformant plants as well as Col-0 grown on plates without selection were transplanted onto Sunshine Mix #1 soil and grown as described above.

### *n*-Gram Classification

Each putative repression motif was broken into all 2-, 3-, and 4-grams, and the number of each of these *n*-grams was tallied within each quartile of the constructs organized by repression strength. In order to qualify as a “strong” *n*-gram, an *n*-gram must be at least 2× over-represented in the strongest quartile of the constructs and under-represented in the weakest quartile. To qualify as “weak”, the opposite must be true: 2× over-representation in the weakest quartile of repression constructs and some amount of under-representation in the strongest quartile. To qualify as a “moderate” *n*-gram, an *n*-gram must not be over- or under-represented by more than 25% in any of the four quartiles.

### Microscopy

*Arabidopsis* floral stem trichomes were imaged using a Leica MZ16F stereomicroscope equipped with an Infinity 3 real-color camera (Teledyne Lumenera) with image capture into Infinity Analyze software.

### RTqPCR

Total mRNA was extracted using an E.Z.N.A. plant RNA kit (Omega Biotek) using on-column DNase digestion, and cDNA synthesis was achieved with an SSIV Vilo IV kit using random hexamers (Thermo Fisher). Quantitative PCR was performed using a CFX96 real-time thermocycler (Bio-Rad) programmed for detection of SYBR intercalating dye with the following temperature programming: 95 °C for 3 min, then 95 °C for 30 s, 60 °C for 45 s repeated 34 times, then a gradual increase from 65 to 95 °C at 0.5 °C/min to generate melt curves. Sso-Advanced Universal SYBR Green Supermix (Bio-Rad) was used for qPCR amplification. Primer sequences are available in Table S2. Normalized relative expression was calculated using the ΔΔCq method and normalized by setting the average level of amplification in the wild-type samples equal to 1.

### Data Analysis

Data were recorded in Google Sheets, and all analyses were performed using Python as implemented in Google Collab. Figures were assembled in Google Drawings.

## Data Availability

A Python notebook to run all analyses and produce all figures in this Letter is available through GitHub: https://github.com/KaseyMarkel/Plant-Transcriptional-Repression-Toolkit/tree/main. Annotated plasmid maps for repression motifs and our reporter plasmid are also available through the JBEI registry: https://public-registry.jbei.org/.

## Supporting Information Available

Natural EAR repressor data (Table S1); primers used for RTqPCR (Table S2); *Arabidopsis* stable line TF-repressor fusion RTqPCR data (Table S3); *n*-grams associated with strong, medium, and weak repression in natural EAR dataset (Table S4); SynEAR repressor data (Table S5); non-EAR and EAR fusion repressor data (Table S6); tandem SRDX data (Table S7); normalized merged averaged data for all repressors characterized in this study (Table S8) (XLSX)
Figures S1–S5 (PDF)
