# Supplementary material for: Design and Characterization of a Transcriptional Repression Toolkit for Plants
Source: ACS Synth Biol. 2024 Sep 24;13(10):3137–43. doi: 10.1021/acssynbio.4c00404 (PMC11494698; doi:10.1021/acssynbio.4c00404)
Supplement: Supplementary file 2 — sb4c00404_si_002.pdf [file sb4c00404_si_002.pdf]

Supplemental figures to accompany the manuscript:

Design and characterization of a transcriptional repression toolkit for plants

Kasey Markel[1,2,3], Jean Sabety[4], Shehan Wijesinghe[4], Patrick M. Shih[1,2,3,5,6]*

1 Department of Plant and Microbial Biology, University of California, Berkeley, Berkeley, CA, USA 94720
2 Feedstocks Division, Joint BioEnergy Institute, Emeryville, CA, USA
3 Environmental Genomics and Systems Biology Division, Lawrence Berkeley National Laboratory, Berkeley, CA, USA 94608
4 Department of Plant Biology, University of California, Davis, California, USA 95616
5 Joint Genome Institute, Lawrence Berkeley National Laboratory, Berkeley, CA, USA 94720United States
6 Innovative Genomics Institute, University of California, Berkeley, CA, USA 94720

*corresponding author email address: pmshih@berkeley.edu

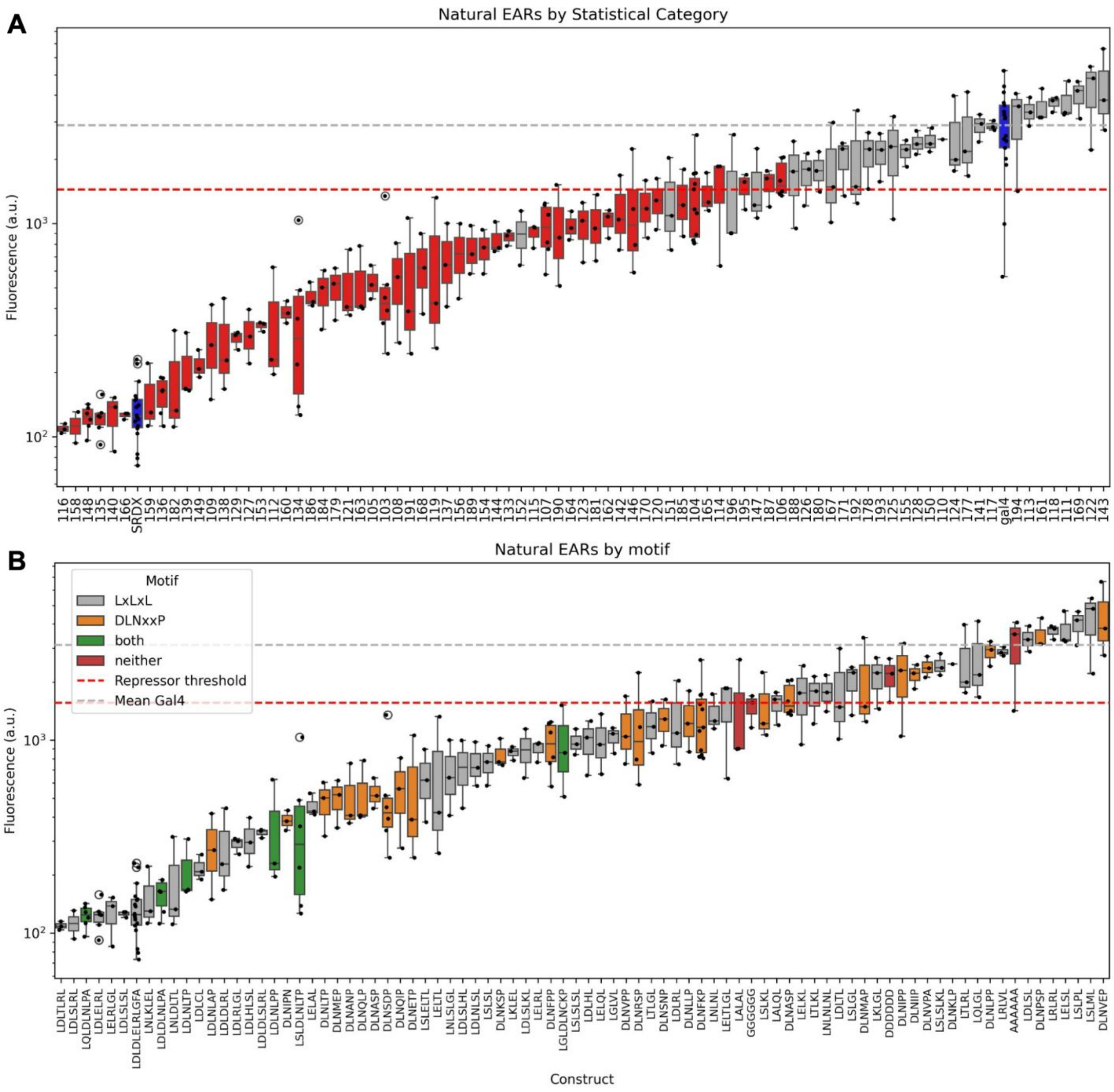

Figure S1: Natural EAR library plotted with additional characteristics. A) EAR library plotted with a statistical significance threshold difference from Gal4 rather than a mean repression difference. EAR constructs are colored red if the mean GFP fluorescence is lower than that of Gal4 as determined by a Mann-Whitney U test with a threshold of $p = 0.05$. Gal4 and SRDX are highlighted in blue. B) EAR library data plotted by whether the construct contains the classic EAR motifs LxLxl, DLNxxP, both, or neither. Boxes indicate 25th, 50th, and 75th percentile, raw data are plotted as points.

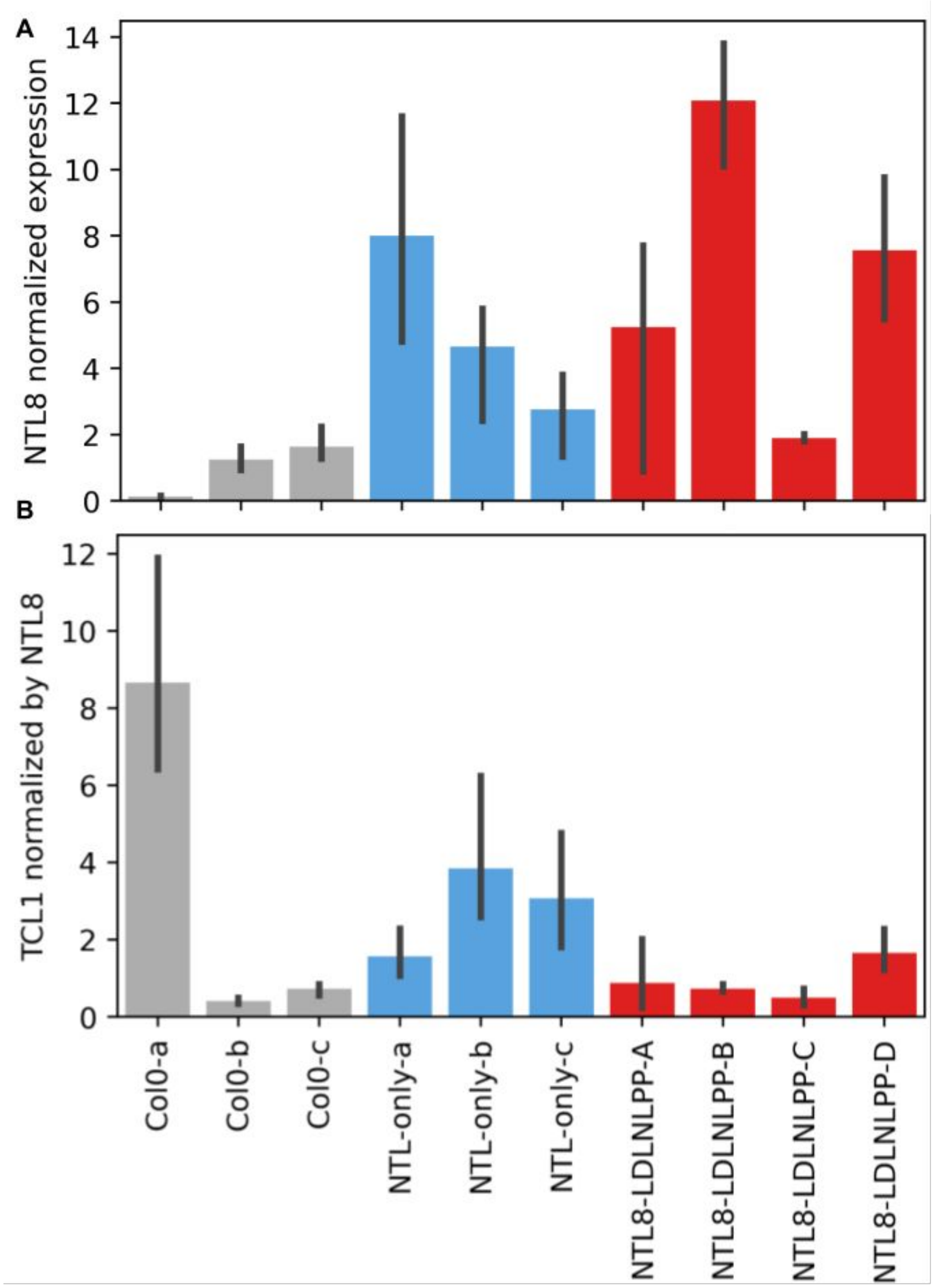

Figure S2: Variance in transgenic NTL8 expression partially explains variance between transgenic lines. A) RTqPCR data for the expression level of NTL8 normalized by Ef1α, with the average Col-0 expression set to 1. The primers bind in the middle of the NTL8 CDS, so this measures native NTL8 expression as well as expression of both transgenic constructs. B) TCL1 expression normalized by Ef1α and then further normalized by NTL8 (itself also normalized by Ef1α). This double-normalization reveals the relationship between the expression strength of the transgenic NTL8 constructs and the effect on TCL1 expression. Primers are available in Table S6,

data are available in Table S7. Error bars indicate the SEM.

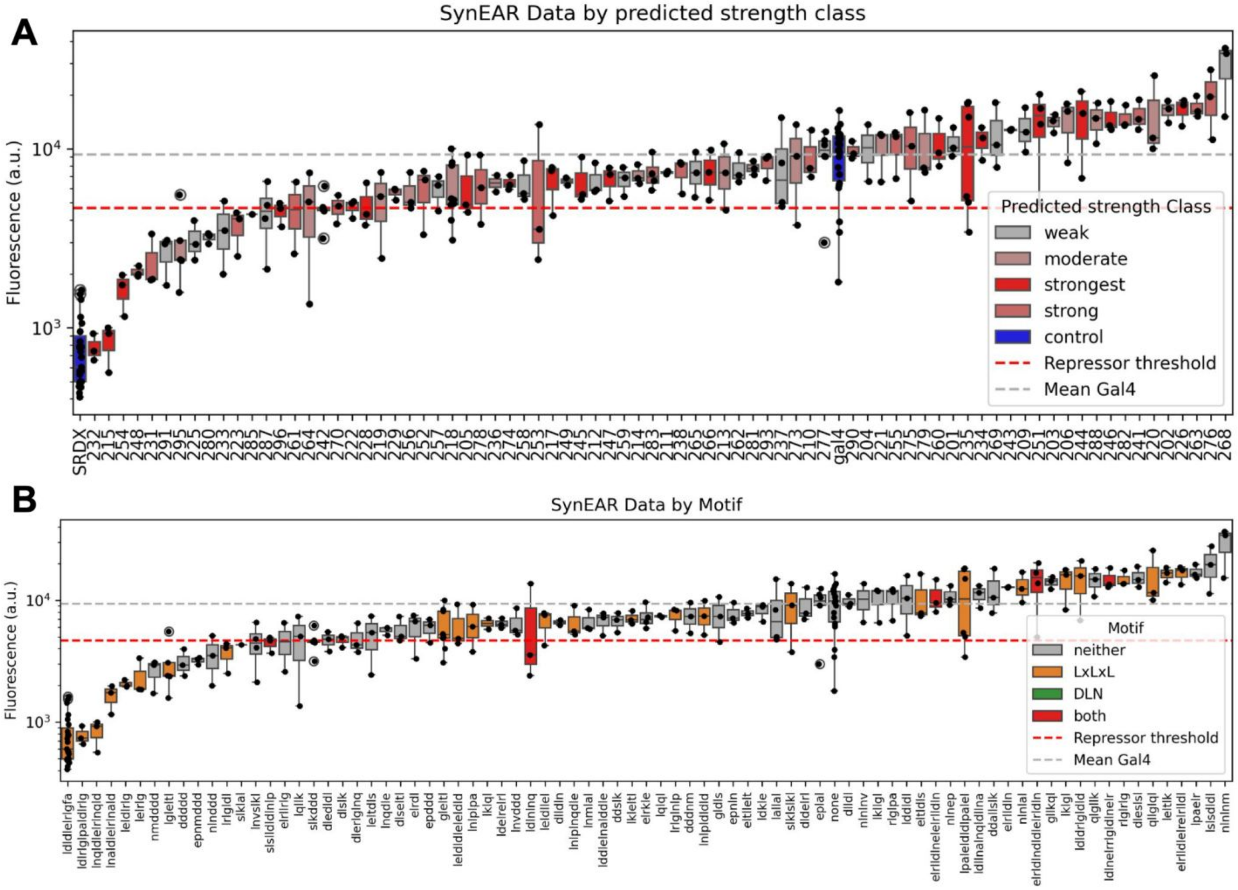

Figure S3: SynEAR library plotted with additional characteristics. A) EAR library plotted with a statistical significance threshold difference from Gal4 rather than a mean repression difference. SynEAR constructs are colored red if the mean GFP fluorescence is lower than that of Gal4 as determined by a Mann-Whitney U test with a threshold of $p = 0.05$. Gal4 and SRDX are highlighted in blue. B) EAR library data plotted by whether the construct contains the canonical EAR motifs LxLxl, DLN, both, or neither. No SynEARs contained the canonical motif DLNxxP, so we here substitute the looser restriction of only DLN for better visualization of near-matches. Boxes indicate 25th, 50th, and 75th percentile, raw data are plotted as points.

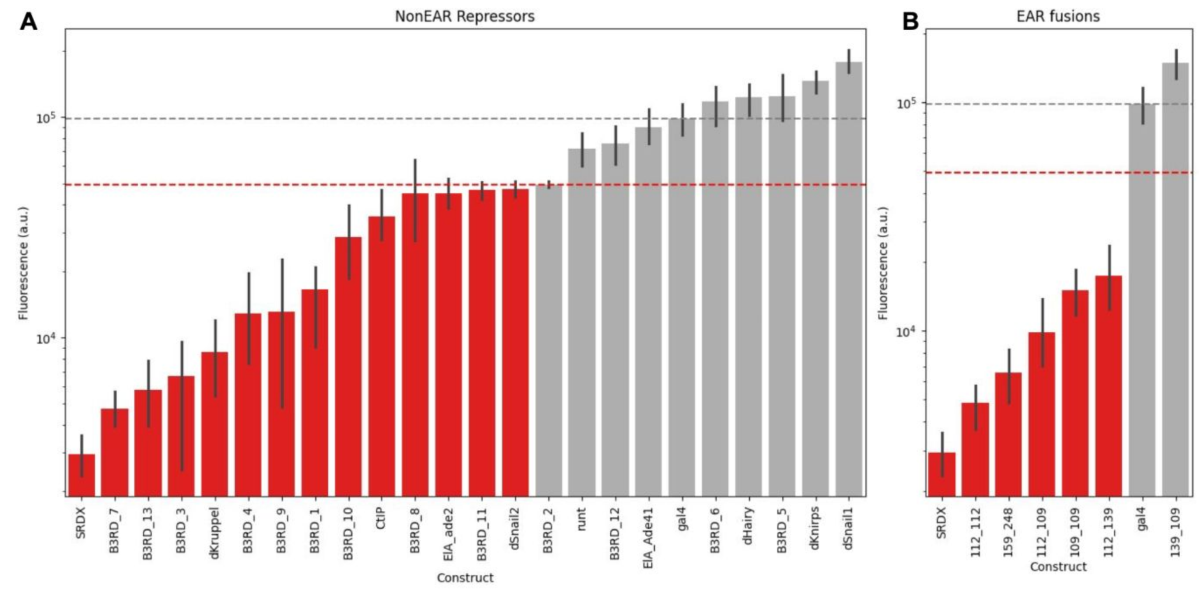

Figure S4: Non-EAR repressors and EAR fusion repressors. A) Trans-regulatory activity of repressor constructs generated from reported transcriptional repressors across Eukaria. Data and sequence information are available in Supplemental Table 4. B) Trans-regulatory activity of fusions of EAR and SynEAR repressors (EARs have ID numbers between 100 and 196, SynEARs have ID between 200 and 296, all sequence compositions and data are available in Supplemental Table 1 and Supplemental Table 3 for EARs and SynEARs respectively). Error bars indicate the SEM.

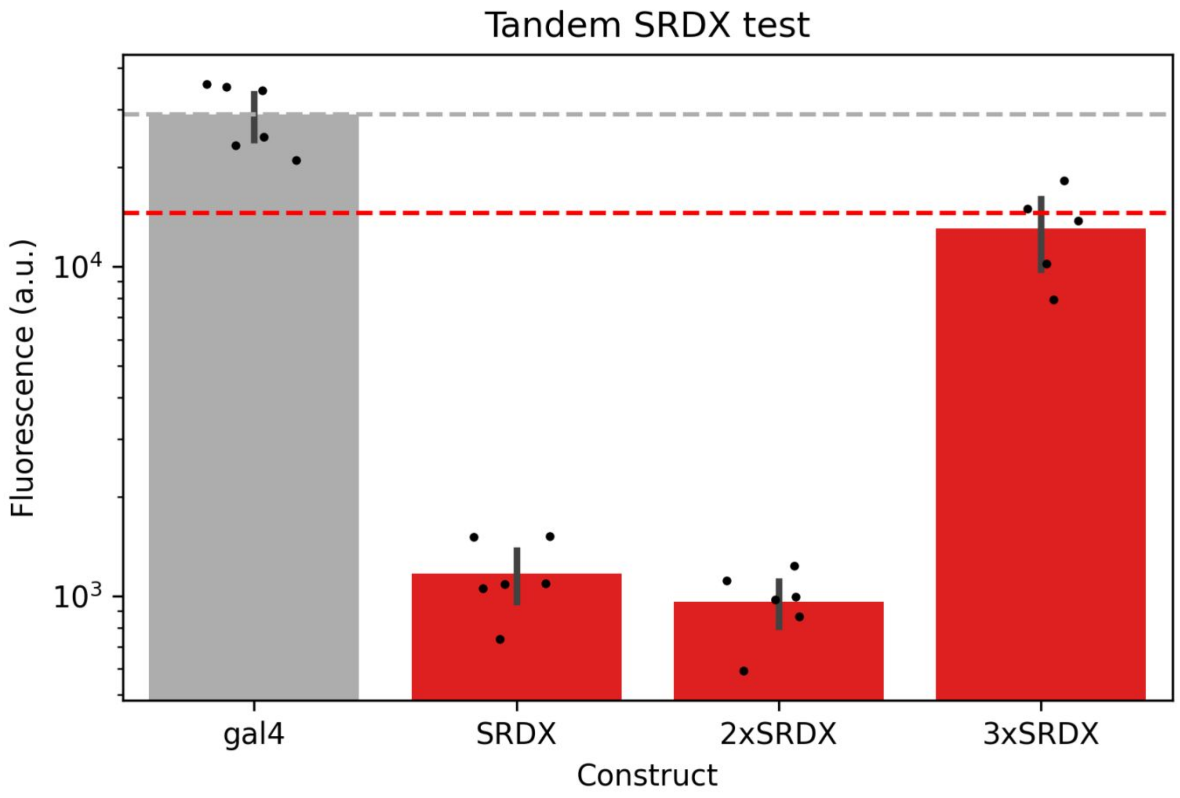

Figure S5: Concatenation of SRDX reduces repression strength. Reporter fluorescence for gal4 and 1x, 2x, and 3x SRDX. Error bars indicate the SEM, raw data are plotted as points

List of additional supplemental files

Table S1: Natural EAR repressor data

Table S2: Primers used for RTqPCR

Table S3: Arabidopsis stable line TF-repressor fusion RTqPCR data

Table S4: Ngrams associated with strong, medium, and weak repression in natural EAR dataset

Table S5: SynEAR repressor data

Table S6: Non-EAR and EAR fusion repressor data

Table S7: Tandem SRDX data

Table S8: Normalized merged averaged data for all repressors characterized in this study

Code: A Python notebook to run all analyses and produce all figures in this manuscript is available through Github: https://github.com/KaseyMarkel/Plant-Transcriptional-Repression-Toolkit/tree/main
